# 

**Published:** 1892-01

**Authors:** 


### CONTENTS—JANUARY, 1892.—VOL. VII, No. 7.

ORIGINAL COMMUNICATIONS—
Is Syphilis Transmitted Directly from the Father to the Child? By C. O. Weller, M. D. .... 233
Discussion on Syphilis .... 241
The Value of the Microscope in Diagnosis and Treatment of Tuberculosis. By J. S. Price, M. D. .... 243
Lucillia Macellaria—"Texas Screw Worm." By J. F. Eaves, M. D. .... 247
CORRESPONDENCE—
Can Men Have Milk Leg? Letter from Grace Danforth, M. D., Chicago .... 248
SOCIETY NOTES—
Discussion on Fevers and Other Subjects, by Austin District Medical Society .... 250
Discussion on Dr. Parker's Paper—Enteritis .... 254
EDITORIAL—
The Asylum Tragedy .... 257
Account of the Killing of Supt. Reeves .... 260
Dr. Reeves' Biography .... 263
Give It a Name .... 264
The New Superintendent .... 265
A Private Hospital for Austin .... 266
MEDICAL NEWS AND MISCELLANY .... 267
PUBLISHER'S NOTES .... 272

